# Supplementary material for: Loneliness amongst Low-Socioeconomic Status Elderly Singaporeans and its Association with Perceptions of the Neighbourhood Environment
Source: Int J Environ Res Public Health. 2019 Mar 18;16(6):967. doi: 10.3390/ijerph16060967 (PMC6466029; doi:10.3390/ijerph16060967)
Supplement: Supplementary file 1 [file ijerph-16-00967-s001.pdf]

Supplementary Table S1: Principal components analysis of the 17-item modified Neighbourhood Environment Walkability Scale Abbreviated (NEWS-A).

Original Subscales of the NEWS	Principal Components derived from Factor Analysis	
	Perceptions of Neighbourhood Safety and Convenience (45.2% of Variance)	Perceptions of Neighbourhood Physical Environment (10.60% of Variance)
**Crime safety (7 items)**		
Presence of litter/trash	0.345	0.676
Lighting/signage in neighbourhood streets	0.303	0.616
Presence of pedestrians/people on the street	0.341	0.637
Observed interaction (e.g., conversation) between pedestrians on the street	0.678	−0.099
Perceived crime rate in neighbourhood	0.728	−0.112
Perceived safety when walking around neighbourhood by day	0.818	−0.134
Perceived safety when walking around neighbourhood by night	0.730	−0.108
**Land use access (2 items)**		
Physical barriers in neighbourhood (e.g., uneven ground, steps)	0.298	0.663
Easy to walk to bus & train stations	0.795	−0.043
**Land use diversity (8 items) Convenience to access the following amenities:**		
Grocery store (provision shop, wet market, supermarket)	0.857	−0.131
Eating place (hawker centre, coffee shop)	0.729	−0.063
Financial services (bank/ATM/post office)	0.761	0.031
Religious institution (e.g., temples, churches)	0.706	−0.071
Dispensaries (pharmacies, medicinal shops)	0.845	−0.128
Medical services (clinics/dentals)	0.863	−0.111
Community activity centre (e.g., community club, senior activity centre)	0.787	−0.097
Recreational areas (e.g., park, swimming pools, gym or fitness facility)	−0.080	−0.030

Factor analysis of the 17 NEWS items produced 2 principal components, summarised as *"perceptions of neighbourhood safety and convenience"* and *"perceptions of neighbourhood physical environment"*. Most of the subscales related to perceived neighbourhood safety (e.g., perceived crime rate in neighbourhood, perceived safety when walking around) and perceived neighbourhood convenience (availability of amenities, such as convenient access to shops, eating places, medical services, and recreational areas) loaded onto the first principal component, while subscales related to the physical environment (e.g., presence of litter, lighting/signage, physical barriers such as uneven ground or steps) loaded onto the second principal component.
